# *In Vitro* Profiling of Antitubercular Compounds by Rapid, Efficient, and Nondestructive Assays Using Autoluminescent Mycobacterium tuberculosis

**DOI:** 10.1128/AAC.00282-21

**Published:** 2021-07-16

**Authors:** Gauri S. Shetye, Kyung Bae Choi, Chang-Yub Kim, Scott G. Franzblau, Sanghyun Cho

**Affiliations:** a Institute for Tuberculosis Research, College of Pharmacy, University of Illinois at Chicago, Chicago, Illinois, USA; b Department of Biomodulation, MJ Bioefficacy Research Center, Myongji University, Yongin, Gyeonggido, Korea

**Keywords:** *Mycobacterium tuberculosis*, autoluminescence, MIC, minimum bactericidal concentration, time-kill curves, intracellular activity, postantibiotic effect, *in vitro* profiling, drug discovery

## Abstract

Anti-infective drug discovery is greatly facilitated by the availability of *in vitro* assays that are more proficient at predicting the preclinical success of screening hits. Tuberculosis (TB) drug discovery is hindered by the relatively slow growth rate of Mycobacterium tuberculosis and the use of whole-cell-based *in vitro* assays that are inherently time-consuming, and for these reasons, rapid, noninvasive bioluminescence-based assays have been widely used in anti-TB drug discovery and development. In this study, *in vitro* assays that employ autoluminescent M. tuberculosis were optimized to determine MIC, minimum bactericidal concentration (MBC), time-kill curves, activity against macrophage internalized M. tuberculosis (90% effective concentration [EC_90_]), and postantibiotic effect (PAE) to provide rapid and dynamic biological information. Standardization of the luminescence-based MIC, MBC, time-kill, EC_90,_ and PAE assays was accomplished by comparing results of established TB drugs and two ClpC1-targeting TB leads, ecumicin and rufomycin, to those obtained from conventional assays and/or to previous studies. Cumulatively, the use of the various streamlined luminescence-based *in vitro* assays has reduced the time for comprehensive *in vitro* profiling (MIC, MBC, time-kill, EC_90,_ and PAE) by 2 months. The luminescence-based *in vitro* MBC and EC_90_ assays yield time and concentration-dependent kill information that can be used for pharmacokinetic-pharmacodynamic (PK-PD) modeling. The MBC and EC_90_ time-kill graphs revealed a significantly more rapid bactericidal activity for ecumicin than rufomycin. The PAEs of both ecumicin and rufomycin were comparable to that of the first-line TB drug rifampin. The optimization of several nondestructive, luminescence-based TB assays facilitates the *in vitro* profiling of TB drug leads in an efficient manner.

## INTRODUCTION

Tuberculosis (TB) is among the top 10 causes of death worldwide (1). Over time, selection of Mycobacterium tuberculosis strains that resist the action of most first- and/or second-line TB drugs has yielded multidrug resistant (MDR) and extremely drug resistant (XDR) variants (2). The prevalence of disease due to drug-sensitive (DS) TB and the increase in the rate of MDR/XDR cases has warranted an escalation in the drug discovery effort (3). Renewed interest in TB has augmented the discovery of novel targets and new chemical entities (NCEs) (3–8).

The hits identified through traditional target-based biochemical assays against M. tuberculosis have a high attrition rate during early discovery (9). This is primarily because hits identified through such target-based assays may have strong *in vitro* target binding but could lack the ability to achieve effective intracellular concentrations, hence exhibiting poor antibacterial activities (10, 11). In contrast, hits identified through whole-cell-based screening assays have a higher chance of generating viable lead compounds (12, 13). Improving upon these established older whole-cell-based assays (WCBAs) can further facilitate the expedition of TB drug discovery (14). There are several WCBAs that are more commonly used for profiling antitubercular activity of NCEs, which include MIC, minimum bactericidal concentration (MBC), time-kill curves, activity against macrophage internalized M. tuberculosis (90% effective concentration [EC_90_]), and post antibiotic effect (PAE) (15).

The single most commonly used microplate-based method for M. tuberculosis MIC determination is using alamarBlue or its active ingredient, resazurin, a nonfluorescent dye that when reduced by the reductive environment created by viable bacterial cells generates the pink, highly fluorescent compound, resorufin (16). Previously, both firefly luciferase (FFluc) and the bacterial luciferase (LuxAB) of Vibrio harveyi have been successfully used as reporters of bacteria cell viability (17). The shortcoming of externally adding the substrate/cofactor to the assay plate when using FFluc and LuxAB was circumvented by deploying autobioluminescence M. tuberculosis strains that express the whole Lux operon (*luxCDABE*) (18, 19). Apart from MIC determination, autobioluminescent M. tuberculosis has also been used for the quantification of MBC and EC_90_ (19–26).

Clinically, drug dosages that fail to achieve complete sterilization can yield resistant M. tuberculosis mutants, and therefore knowing whether a new compound class is bacteriostatic or bactericidal during early-stage discovery is important for prioritization (27, 28). Traditionally, MBC is determined by CFU, wherein the bacterial count after about 7 days exposure in liquid culture is compared to the bacterial count prior to compound exposure. Because colony formation requires approximately 3 weeks, the typical turnaround time for an MBC determination is 1 month. Unlike MIC determination, where various spectroscopic readouts like absorbance, fluorescence, and luminescence can be used for measuring bacterial growth inhibition, the same is more challenging for MBC, which requires the quantification of low levels of the initial bacterial count by instruments with high sensitivity. Previously, two separate studies have developed and progressed the use of autobioluminescent M. tuberculosis in MBC determination (20, 21). Both studies (20, 21) used a similar approach where the bacterial culture was exposed to an antibiotic for a period of time and then diluted and regrown to allow the autobioluminescent M. tuberculosis to attain the method’s limit of quantitation (LOQ) relative light units (RLU) threshold, a regrowth period defined as “time to positivity” (TTP) by the latter.

The ability of M. tuberculosis to survive within alveolar macrophages makes antibiotic action more exacting and necessitates a prolonged therapy time (29, 30). From a drug discovery standpoint, it is important to identify NCEs that not only possess antimycobacterial activity against extracellular M. tuberculosis but also against intracellular (macrophage internalized) M. tuberculosis (31, 32). Therefore, an *in vitro* WCBA that evaluates the activities of new compounds against macrophage-internalized M. tuberculosis in a concentration- and time-dependent manner can aid in prioritization for *in vivo* evaluation (20, 33). Previously, Andreu et al. established the use of bioluminescence for quantifying intracellular activity of some TB drugs against the M. tuberculosis within macrophages (20).

PAE, the suppression of bacterial growth after a short exposure to an antibiotic, has been defined as the difference in time (h) taken by antibiotic-exposed and control (no drug) bacterial cultures to increase by 1 log_10_ unit (34). This parameter is also important for establishing dosing and gathering crucial information on target vulnerability (9). Similar to MBC and EC_90_ assays, the preferred method of quantitation for PAE has been viable cell counting by determination of CFU (35). The use of radiometric Bactec culture medium and the subsequent measurement of labeled ^14^CO_2_ has also been reported for quantitation of PAE (34, 36).

Enumerating CFU or viable cell counting is the most popular quantification method deployed in M. tuberculosis-related killing (MBC), intracellular activity (EC_90_), and PAE assays (34, 37, 38). While these parameters can be accurately determined through viable cell count enumeration, this process is very labor-intensive and time-consuming, thereby typically limiting the number of evaluated compounds.

Despite the significance of these WCBAs in anti-TB drug discovery, most remain static and/or low throughput. In this study, we describe the optimization of efficient, rapid, and high-throughput capable, luminescence-based assays (LBAs) for determining MIC, MBC, time-kill curves, EC_90_, and PAE. The standardization of these LBAs was accomplished by direct comparison with results from traditional assays and/or previous reports. In this study, the H37Rv (ATCC 27294) strain of M. tuberculosis was transformed with the pMV306G13+Lux plasmid (19) to yield transformed colonies that are autobioluminescent due to the ability to express the *luxABCDE* operon.

## RESULTS

### Autoluminescence-based MIC assay versus microplate alamarBlue assay.

We compared the results from luminescence-based MIC assay (LMICA) with the microplate alamarBlue assay (MABA). A strong overall correlation (*R*^2^ = 0.93) was observed between MABA and LMICA MIC values for 10 TB drugs having different modes of action and two anti-M. tuberculosis leads (ecumicin and rufomycin) (Table 1).

**TABLE 1:** MICs of TB drugs and two emerging leads (ecumicin and rufomycin) determined by LMICA and MABA

Drug name	Mode of action/target	MIC (μM) (±SD)^*a*^	
		LMICA	MABA
Rifampin	RNA polymerase/RpoB	0.041 (0.005)	0.060 (0.01)
Moxifloxacin	DNA replication/GyrA	0.13 (0.02)	0.24 (0.04)
Linezolid	Protein synthesis/rRNA	1.7 (0.23)	1.7 (0.10)
Streptomycin	Protein synthesis/30S ribosome	0.12 (0.04)	0.12 (0.02)
Capreomycin	Protein synthesis/70S ribosome	0.53 (0.07)	0.70 (0.03)
Isoniazid	Cell wall synthesis/InhA	0.24 (0.08)	0.30 (0.05)
Ethambutol	Cell wall synthesis/EmbA	1.3 (0.38)	2.0 (0.41)
Pretomanid	Mycolic acid biosynthesis and NO production	0.46 (0.01)	0.49 (0.02)
Bedaquiline	Energy metabolism/AtpE	0.028 (0.01)	0.021 (0.01)
Clofazimine	Energy metabolism/NDH-2	0.010 (0.02)	0.014 (0.02)
Ecumicin	Proteolysis/ClpC1	0.074 (0.002)	0.081 (0.02)
Rufomycin	Proteolysis/ClpC1	0.010 (0.001)	0.032 (0.012)

a The standard deviation is derived from three independent experiments.

### MBC determined by luminescence versus CFU.

A compound is generally considered bactericidal if it’s MBC/MIC is at least 4-fold or less (<4); otherwise, it is regarded as bacteriostatic (>4) (15, 39). In this study, MBC was defined as the lowest concentration of a compound that reduces the initial bacterial inoculum by 99% (or 2 log) (38).

**FIG 1:**
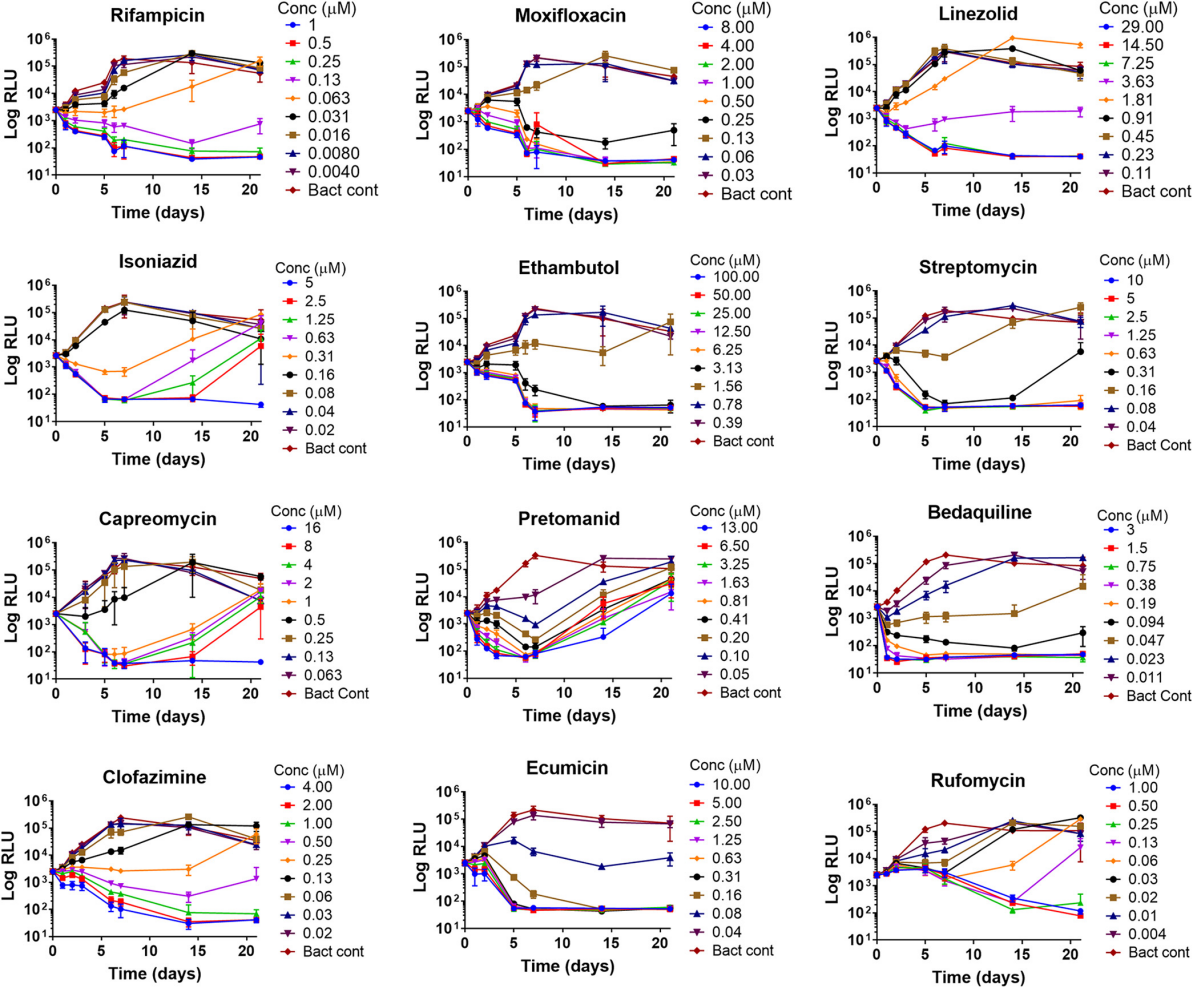
Time-kill curves (relative luminescence unit [RLU] versus time) of 10 TB drugs and two TB leads, ecumicin and rufomycin, at various concentrations. Kill curve of each drug includes a plot for bacterial control with no drug added. Standard deviation (SD) is from three independent experiments. Background RLU on day 21 is 50 (±6).

Evaluation of the luminescence-based MBC assay (LMBCA) was achieved by comparing MBC values for 10 TB drugs and two emerging TB leads, ecumicin and rufomycin, to a CFU measurement (Table 2). For the LMBCA, the bacterial luminescence was measured at multiple time points (up to day 21) to obtain both MBC and time-kill information (Fig. 1). Mean MBC values of 8 of the 10 TB drugs varied by less than 2-fold between the assays. For clofazimine and linezolid, there was at least a 2- or 3-fold difference in measured MBCs via the two methods, the actual magnitude of which could not be determined at the concentrations that were tested. Interestingly, the two ClpC1 inhibitors, ecumicin and rufomycin, exhibited unintuitive trends in MIC and MBC. While the MIC of rufomycin was 7-fold lower than ecumicin (Table 1), on day 7, ecumicin showed strong bactericidal activity while rufomycin was bacteriostatic at the tested concentrations (Table 2 and Fig. 1).

**TABLE 2:** MBCs from LMBCA or CFU assays for 10 TB drugs and two emerging (ecumicin and rufomycin) TB leads

Drug name	MBC (μM) (±SD)^*a*^	
	Via luminescence	Via CFU
Rifampin	0.6 (0.2)	0.4 (0.06)
Moxifloxacin	0.91 (0.25)	0.753 (0.4)
Linezolid	9.6 (2.26)	>29^*b*^
Streptomycin	0.3 (0.05)	0.37 (0.1)
Capreomycin	1.3 (0.4)	1.7 (0.9)
Isoniazid	0.60 (0.08)	0.46 (0.21)
Ethambutol	9.2 (2.7)	7.9 (4.0)
Pretomanid	0.63 (0.05)	0.5 (0.17)
Bedaquiline	0.13 (0.04)	0.16 (0.04)
Clofazimine	1.8 (0.19)	>4^*b*^
Ecumicin	0.22 (0.05)	0.10 (±0.03)
Rufomycin	>1^*c*^	>1^*b*^

a The standard deviation is from three independent experiments.

b The MBC was greater than the highest tested concentration in all three experiments.

c Rufomycin showed time dependency in bactericidal activity, 99% reduction in luminescence and CFU was not achieved on day 7, and its bactericidal activity was apparent on day 21 (MBC = 0.5 μM [±0.01]) in luminescence assay.

### Luminescence-based MBC assay can be easily adapted to generate time-kill curves.

The *in vitro* pharmacokinetic-pharmacodynamic (PK-PD) effect of an antibiotic against a microbe as a function of a drug concentration and/or the time of exposure can be facilitated by plotting time-kill curves at different concentrations. Since the luminescence-based MBC assay described above is nondestructive, the luminescence of the microtiter plate was measured at additional time points up to day 21, allowing measurement of kill kinetics. In addition, since within a single microtiter plate the drug was serially diluted, its concentration-dependent killing capacity could also be assessed. RLU versus time graphs were plotted to obtain dose/time-kill curves (Fig. 1). Time-kill curves can potentially yield the following three trends with increasing time of exposure: (i) no change in drug activity, (ii) decrease in drug activity (MBC value increases), and (iii) increase in drug activity (MBC value decreases) (28). Over time (21 days), MBC values decreased for rifampin, moxifloxacin, ethambutol, linezolid, clofazimine, ecumicin, and rufomycin and remained unchanged for bedaquiline and streptomycin (Fig. 1; see also Table S1 in the supplemental material). The observed increase in MBC values (Table S1) for isoniazid, capreomycin, and pretomanid is likely due to the emergence of resistant mutants (28). Bactericidal activities of rifampin, moxifloxacin, clofazimine, streptomycin, and pretomanid were both time and concentration dependent (40). The antimycobacterial activities of linezolid, isoniazid, ethambutol, ecumicin, and rufomycin were mainly time dependent (28, 37, 38), whereas bedaquiline was primarily concentration dependent (41).

### Assessment of intracellular anti-TB activity using autoluminescent M. tuberculosis.

**FIG 2:**
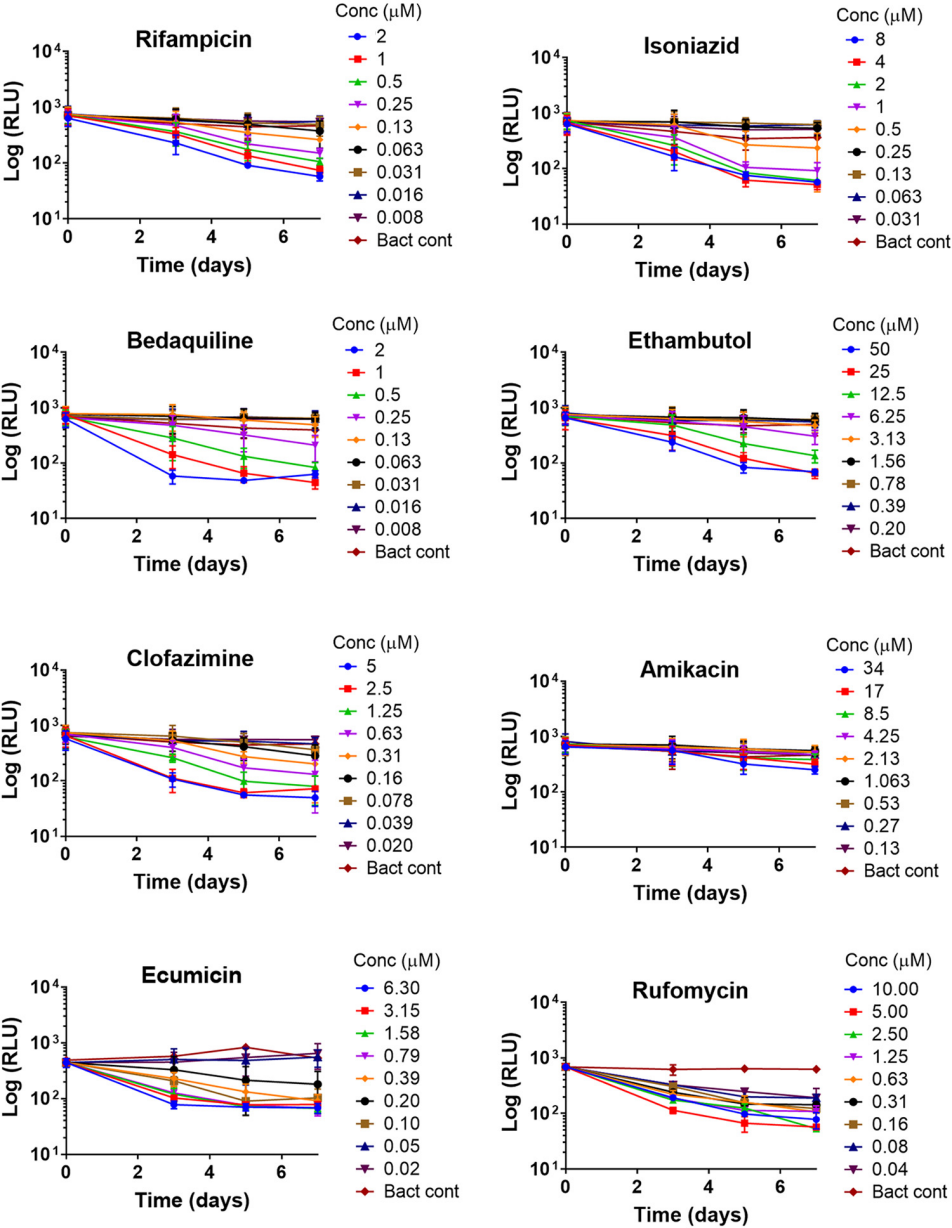
Time-dependent anti-TB activity of six established TB drugs and two TB leads (ecumicin and rufomycin) against intracellular M. tuberculosis evaluated by measuring bacterial autoluminescence (RLU) over a period of 7 days. Background RLU on day 7 is 41 (±8).

Next, we evaluated an *in vitro* luminescence-based assay to assess the intracellular antimicrobial activities (EC_90_) of compounds against macrophage-internalized M. tuberculosis. The murine macrophage cell line J774 was infected with autoluminescent M. tuberculosis strain H37Rv_LuxABCDE. Intracellular activity of the compounds was determined after 7 days of incubation with subsequent luminescence measurements on days 3, 5, and 7 (Fig. 2). At the end of the EC_90_ experiment (on day 7), resazurin dye was added, and fluorescence was measured (after 3 h) to enable the counting of viable J774 cells. This enabled the determination of compound cytotoxicity against infected macrophages at the tested concentrations (Table 3). Amikacin, which is known to be active against extracellular but not against intracellular M. tuberculosis, was used as a control, and the inability to obtain an EC_90_ endpoint (Table 3) is consistent with its predicted antimycobacterial behavior (42). The EC_90_ values of 6 TB drugs and 2 TB leads, ecumicin and rufomycin, were obtained via the luminescence-based assay and then compared to their literature reported (31, 33, 37, 38, 43, 44) values (from either CFU-based or high-content imaging) (Table 3). Overall, there was an agreement between the day 7 EC_90_s obtained and literature reported values for the following 6 established TB drugs: rifampin, isoniazid, ethambutol, clofazimine, bedaquiline, and amikacin (Table 3). Slight variation in EC_90_ values could be due to the use of differing cell lines and/or dissimilar end points, i.e., different post drug exposure days used for calculating EC_90_. For example, the literature reported EC_90_ values of rifampin, isoniazid, and amikacin were obtained via high-content imaging on day 5 (33). For rifampin, isoniazid, and amikacin, the head-to-head EC_90_ comparison on day 5 is generally consistent between both luminescence-based and high-content imaging.

**TABLE 3:** RLU and literature reported EC_90_ values of six established TB drugs and two TB leads (ecumicin and rufomycin) against J774-internalized M. tuberculosis (H37Rv)^*a*^

Drug name	Intracellular activity, EC_90_ (μM) (±SD)^*b*^			Literature reported EC_90_ (μM) (reference)	Toxicity against infected J774^*b*^
	Day 3	Day 5	Day 7		
Rifampin	>2 (60)*	1.55 (0.53)	0.64 (0.08)	2.9 (33)	2 (0)*
Isoniazid	>8 (77)*	1.05 (0.47)	0.52 (0.25)	1.2 (33)	8 (0)*
Ethambutol	>50 (68)*	44 (7.5)	23.84 (2.8)	16.7 (31)	50 (0)*
Clofazimine	19.03 (3.2)	6.69 (2.6)	3.70 (0.93)	∼2.1 (43)	50 (45)*
Bedaquiline	0.89 (0.4)	0.29 (0.14)	0.22 (0.12)	0.10 (44)	2 (7)*
Amikacin	>34 (4)*	>34 (34)*	>34 (49)*	>26 (33)	>34 (0)*
Ecumicin	0.74 (0.51)	0.57 (0.20)	0.28 (0.1)	∼0.12 (38)^*c*^	6.3 (11)*
Rufomycin	>10 (69)*	3.06 (0.10)	1.52 (0.60)	∼0.1 (37)^*c*^	10 (8)*

a Cytotoxicity of each drug against the infected J774 cells on day 7 is also reported. Standard deviation is from three independent experiments.

b An asterisk (*) indicates percent inhibition at the highest tested concentration (μM).

c J774 infected with M. tuberculosis strain Erdman.

### Postantibiotic effect by autoluminescence.

**FIG 3:**
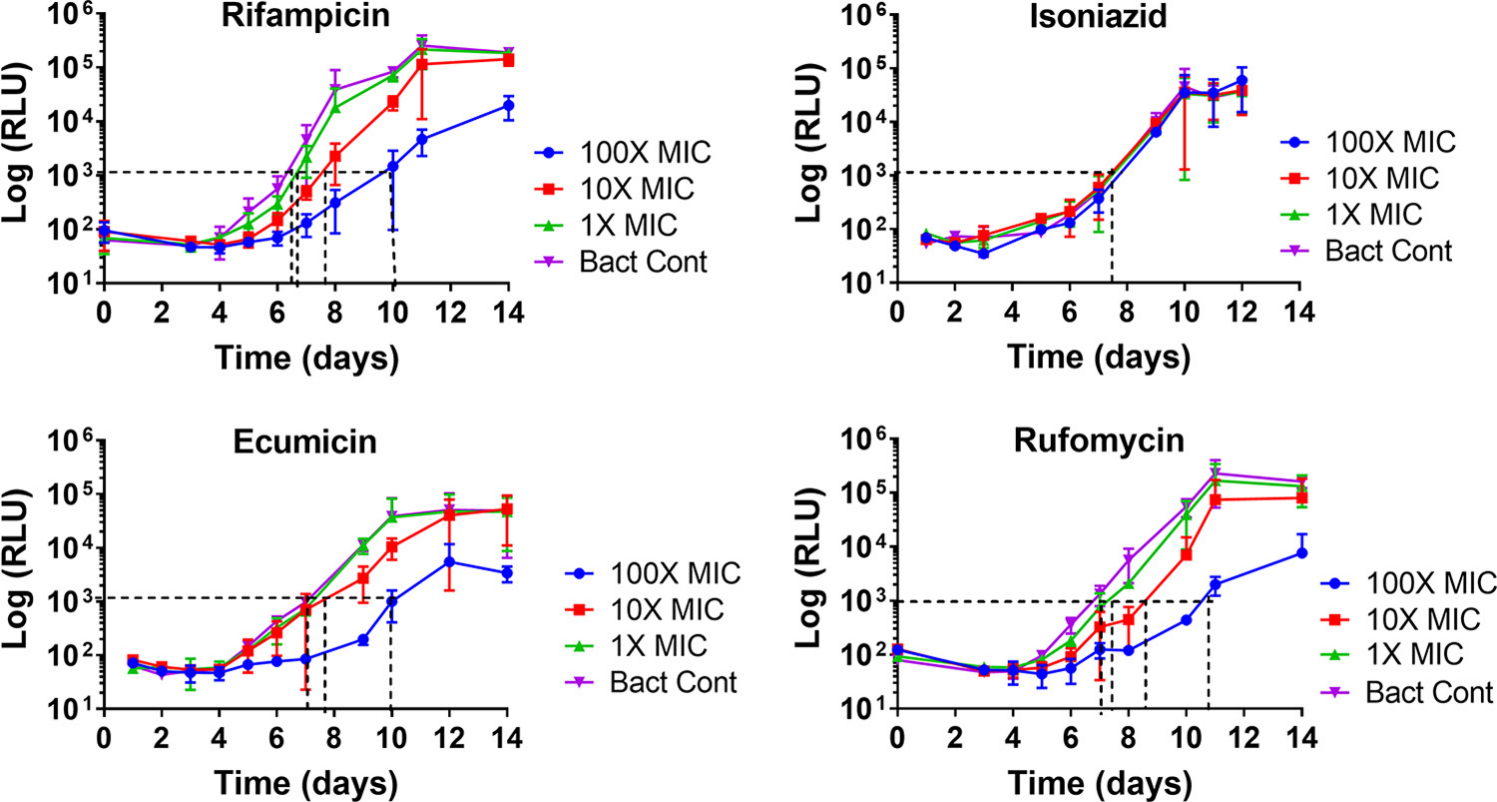
Growth curves of autoluminescent M. tuberculosis strain H37Rv_LuxABCDE after a 3 h exposure to rifampin, isoniazid, ecumicin, and rufomycin at multiple concentrations (1× MIC, 10× MIC, and 100× MIC). Growth of M. tuberculosis H37Rv_LuxABCDE was followed by both CFU and measuring the autoluminescence (RLU). The time interval (*x* axis) between the dotted lines represents PAE in hours at a given concentration. Background RLU on day 14 is 3,000 (±830).

In this study, PAE by luminescence was defined as a difference in time taken by the drug-treated bacterial culture (T) and its corresponding no drug control (C) to register a 1.5-fold increase in log_10_ RLU (see Fig. S1a in the supplemental material). For PAE by RLU, the H37Rv_LuxABCDE culture was incubated with TB drugs at 1× MIC, 10× MIC, and 100× MIC for a period of 3 h, after which the drug-treated bacterial culture was 10-fold serially diluted five times in 7H12 medium (Fig. S1b). Growth of the diluted culture was monitored by RLU over a period of 14 days (Fig. 3). For standardization, it was determined that the PAE values (h) obtained by taking a time difference for the 1.5 log_10_ increase in RLU were comparable to a time difference for 1.0 log_10_ increase in CFU (Table 4). Rifampin exhibited no observable PAE at 1× MIC, but at 10× MIC and 100× MIC, its PAEs were 26 h (RLU) and 30 h (CFU) and 92 h (RLU) and 58 h (CFU), respectively (Table 4). The cell wall biosynthesis inhibitor, isoniazid, exhibited no observable PAE by RLU or CFU even at the highest tested concentration, i.e., 100× MIC (35). Interestingly, both ClpC1 inhibitors, ecumicin and rufomycin, showed strong PAEs that were comparable to those observed for rifampin. Ecumicin had a PAE of 12 h by RLU and 10 h by CFU at 10× MIC and 96 h by RLU and 80 h by CFU at 100× MIC. Rufomycin had a PAE of 40 h by RLU and 48 h by CFU at 10× MIC and 96 h by RLU and 110 h by CFU.

**TABLE 4:** Comparison of post antibiotic effect values (h) obtained via CFU and RLU measurement for two established TB drugs (rifampin and isoniazid) and two emerging TB lead candidates (ecumicin and rufomycin)^*a*^

Drug	MIC (μM)	PAE (h)					
		1× MIC		10× MIC		100× MIC	
		RLU	CFU/ml	RLU	CFU/ml	RLU	CFU/ml
Rifampin	0.04	0	0	26	30	92	58
Isoniazid	0.30	0	0	0	0	0	0
Ecumicin	0.1	0	0	12	10	96	80
Rufomycin	0.02	0	0	40	48	96	110

a The PAE values are obtained from comparing the growth of treated and control bacterial cultures in row F (1:10,000-fold dilution).

## DISCUSSION

While the time and labor advantages of using autoluminescent M. tuberculosis for those *in vitro* drug candidate profiling assays that have continued to rely upon CFU readouts are readily apparent, there are also advantages when compared to other microbroth assays that have largely supplanted CFU-based assays, such as high-throughput primary screening and MICs. The lack of a requirement to add a substrate in the LMICA reduces cost, especially in high-throughput sequencing (HTS) campaigns, as well as provides the option of observing the kinetics of inhibition. It also reduces the total time compared to resazurin-based assays, as the latter requires an additional overnight incubation before measuring fluorescence. In addition to shortening assay time and being HT-capable, the nondestructive endpoint measurement allows the use of the same LMICA microtiter plate for determination of both MBC and time-kill information.

The determination of MBC with M. tuberculosis by CFU is a significantly labor-intensive and time-consuming procedure. In a traditional CFU-based MBC assay for M. tuberculosis, after 7 days exposure to the drug, the bacteria are washed, serially diluted, plated, and CFU enumerated after 3 to 4 weeks. Since this process is very cumbersome, it is often limited to a single time point (usually day 7) measurement. In contrast, the LMBC assay can reveal bactericidal dynamics (both concentration and time dependent) by incubating the same microtiter plate up to 21 days. Previous studies that used autobioluminescent M. tuberculosis for MBC determination have been important in progressing autobioluminescence as a versatile readout alternative to CFU enumeration (20–23, 45, 46). In one such study by Sharma et al. (21), post drug exposure, the bacterial culture was serially diluted to minimize the drug’s inhibitory effect, and then bacterial cells were allowed to regrow to attain an RLU of 1,000 (LOQ). The TTP approach by Sharma et al. was adopted due to the low luminescence sensitivity of the microplate reader, having a signal-to-noise (S/N) of ∼10 at ∼10^6^ CFU/ml. While serial dilution may minimize a drug’s inhibitory effect, it does not completely eliminate any associated PAE, possibly skewing MBC values. In this study, the use of a sensitive luminometer (Centro XS^3^ LB 960; Berthold Technologies), having an S/N of 4 at 5 × 10^3^ CFU/ml, circumvented the need for using the TTP approach. The high sensitivity of the luminometer allowed direct RLU measurement, even at low bacterial concentrations (∼5 × 10^3^ CFU/ml), and therefore eliminating the need for serial dilution.

Both MIC and MBC are static *in vitro* parameters that represent a single-point drug-pathogen relationship, i.e., either at a specific exposure time and/or a specific drug concentration. In contrast, the actual *in vivo* drug-pathogen interaction is more dynamic, with many permutations of drug exposure times and/or concentrations at the effective site (47). Therefore, *in vitro* antibiotic kill curves can give necessary insights into a drug’s overall dynamic killing capacity by projecting a trend in its activity with changes in its concentration and/or exposure time (47). Time-kill curves act as *in vitro* PK-PD models for predictive dose adjustment, synergistic dosage estimation, and complete extinction projection (48). Inability to estimate dosage for complete extinction or inappropriate dosage or incorrect exposure time can lead to failed therapy or emergence of resistant strains (49). We extended the duration of LMBC assay to 21 days to observe time-kill kinetics of 10 TB drugs. Since both MBC and the time-kill capacity of a compound can be obtained via a single LMBC assay, this condensed platform is very efficient. After day 7, there was a slow decrease in bacterial luminescence, and this may be due to the depletion of nutrients in the medium in a 96-well plate as bacterial culture reached lag phase. The LMBC assay was also used for elucidating the killing dynamics of two emerging TB leads, rufomycin and ecumicin, that target the M. tuberculosis ClpC1 proteolysis machinery (38, 50). The time-kill graph (Fig. 1) of rufomycin shows that at concentrations above its MIC, the compound is bacteriostatic by day 7, after which it shows a time-dependent killing response. A traditional CFU-based 7-day MBC assay would have failed to reveal the time-dependent late killing ability of rufomycin. Unlike rufomycin, another ClpC1-targeting cyclic peptide, ecumicin, showed a classical bactericidal dose response (Fig. 1). The mechanistic differentiation in kill kinetics is not surprising given that the binding of these two cyclic peptides to ClpC1 modulates the target differently (37, 38). The determination of MBC on day 7 may capture accurate mechanistic information for most drug-bacteria interactions; however, for M. tuberculosis with a doubling time of ∼20 h, extending the MBC assay up to 21 days can differentiate between true killing and mycobacterial resilience (see Table S1 in the supplemental material).

For TB drug discovery, measurement of the intracellular activities of NCEs through *in vitro* testing can identify leads with a higher probability of success in animal models (51). The luminescence *in vitro* intracellular platform allows for more rapid and expanded compound screening capacity than its CFU counterpart. Here, we optimized the intracellular assay previously described by Andreu et al. (20). Since growth of the H37Rv strain within J774 macrophages remained static (Fig. 2), this assay is unable to distinguish a compound’s bacteriostatic intracellular activity. An added advantage of the *in vitro* luminescence intracellular assay is that at the end of the experiment, the same microtiter plate can also be used for assessing compound cytotoxicity against infected macrophages. We also adapted the nondestructive *in vitro* autoluminescence intracellular assay to give time-dependent information (Fig. 2). Bedaquiline, clofazimine, and ecumicin showed early growth inhibition of internalized M. tuberculosis (EC_90_ obtainable by day 3), whereas, rifampin, ethambutol, and isoniazid showed late growth inhibition (EC_90_ obtainable by day 5 or later) (Table 3 and Fig. 2). The intracellular data (Fig. 2 and Table 3) shows that ecumicin is a relatively faster acting compound than rufomycin (EC_90_ obtainable by day 3 versus day 5, respectively). Both ecumicin and rufomycin exhibited consistent time-kill trends against intracellular and extracellular M. tuberculosis.

Generally, PAE could be attributable to either the physicochemical properties of drug, such as its ability to persist at the binding site, or the pathogen’s inability to quickly repair damaged enzymes or cellular components (52). The PAE of a drug is an important pharmacodynamic parameter for understanding the compound’s internalization, its residence time on the target, target’s vulnerability, and predicting dosing interval (53). Despite its importance, during lead identification, PAEs are generally evaluated for a limited number of promising compounds. The conventional CFU-based PAE assay is labor-intensive, requires a long incubation time, and is not adaptable for real-time data acquisition. Here, we have described an autoluminescence-based PAE assay, which generates real-time data using basic instrumentation (microplate luminometer) available in a biosafety level 3 (BSL3) laboratory. Both RLU and CFU data demonstrated extended and consistent PAE of the RNA polymerase inhibitor rifampin, whereas the cell wall inhibitor, isoniazid, exhibits no apparent PAE. The latter is consistent with previous reports for cell wall inhibitors (36). However, some studies have shown that isoniazid can also exhibit some PAE when the initial drug exposure time is sufficiently long, e.g., 24 h (54, 55). While increasing the drug exposure time can certainly manifest a stronger PAE, a shorter exposure precludes or minimizes significant bacterial cell death. Achieving a standardization that correlates PAE values (h) obtained by taking a 1.5 log_10_ difference in RLU with a corresponding 1.0 log_10_ difference in CFU/ml, we have integrated any growth-related variable. Hence, the use of a 3-h drug exposure time is only for the proof of concept; in theory, any exposure time can be standardized depending upon the scientific requirement. Strong PAEs of two ClpC1 modulators, ecumicin and rufomycin, further confirm the vulnerability of target to such cyclic peptides (9). The acquired PAE of ecumicin and rufomycin will also be useful in predicting dosing within animal models.

In conclusion, these assays provide the tools necessary for the identification and prioritization of new compounds/classes, which have the potential to reduce TB treatment duration. The autoluminescent-based platform is adaptable for one-pot MIC and MBC determination, nondestructive for generating real-time information, and versatile for acquiring concentration and/or time-dependent kill kinetics, intracellular activities, and real-time PAEs of tested compounds against M. tuberculosis in *in vitro* models. The adaptability of the assay to give extracellular and intracellular time-kill data creates an *in vitro* platform that can better predict the dynamic drug-pathogen interaction *in vivo*. The optimized luminescent-based assays were purposeful in profiling the biological activities of two ClpC1 modulators, ecumicin and rufomycin, including the faster killing capacity of ecumicin over rufomycin and their PAEs being comparable to the first-line TB drug, rifampin. We are further evaluating the adaptability of the LMBC assay to assess *in vitro* drug combination effects.

## MATERIALS AND METHODS

### Chemicals and stock solutions.

Rifampin, isoniazid, ethambutol dihydrochloride, capreomycin sulfate, moxifloxacin hydrochloride, clofazimine, streptomycin, and metronidazole were purchased from Sigma-Aldrich (St. Louis, MO, USA). Linezolid, pretomanid (PA-824), and bedaquiline (TMC-207) were received from The Global Alliance for TB Drug Development. Ecumicin was isolated from *Nonomuraea* sp. MJM5123, and rufomycin was isolated from *Streptomyces* sp. strain MJM3502 using previously published chromatographic methods (37, 38, 56, 57). Stocks of all anti-TB agents were prepared in dimethyl sulfoxide (DMSO) at respective concentrations except for isoniazid, ethambutol, and streptomycin, which were prepared in water. All of the stocks were sterilized by filtering through a 0.22 μm syringe filter.

### Strains and media.

M. tuberculosis strain H37Rv (ATCC 27294) was transformed with bacterial luciferase encoding vector pMV306G13+Lux and was used for all of the experiments. Plasmid pMV306G13+Lux was a gift from Brian Robertson and Siouxsie Wiles (Addgene plasmid number 26160) (19). The H37Rv_LuxABCDE strain was grown at 37°C in 7H9 medium (Middlebrook, Difco) supplemented with 0.5% glycerol, 0.05% Tween 80, and 10% oleic acid-albumin-dextrose-catalase (OADC) (Middlebrook, Difco) or in 7H12 medium containing 4.7 g 7H9 broth, 1 g Casitone (Bacto), 5 g bovine serum albumin (BSA), 4 mg catalase, and 5.6 mg palmitic acid for 1 liter medium or on 7H11 agar containing 0.5% glycerol and 10% OADC. To prepare the H37Rv_LuxABCDE bacterial stocks, the culture growing in 7H9 medium was allowed to reach the log growth phase, and then aliquots (1.0 ml each) were passed through 8-μm syringe sterile filters and collected into screw-cap microcentrifuge tubes (size 1.5 ml). Prior to freezing the bacterial stock vials for storage, the luminescence of the filtered culture was measured at different dilution folds (50, 100, 250, 500, and 1,000 dilution folds). The CFU of the bacterial stock was determined by plating on 7H11 agar plates. Depending upon the CFU, the exact dilution fold for that batch of bacterial stocks was decided to get ∼3 × 10^5^ to 5 × 10^5^ cells/ml in the assay well, and luminescence obtained for that dilution fold while freezing stocks was considered as day 0 RLU. The bacterial stock vials were stored frozen at −80°C and thawed prior to use as needed.

### Assay plate preparation for MIC and MBC determination.

The assay plates needed for determination of MIC via luminescence and MABA and MBC via luminescence were prepared in a BSL2 laboratory as follows. The MIC/MBC via luminescence used white opaque 96-well microtiter plates (Thermo Scientific), whereas MIC by MABA used clear 96-well microtiter plates (Thermo Scientific). To avoid the evaporation of medium from test wells, 200 μl of 7H12 medium was pipetted in outer-perimeter wells. To the remaining inner wells, 100 μl of 7H12 medium was then pipetted. An additional 100 μl of 7H12 medium was then added to wells in column 3. Stock solutions of anti-TB compounds were prepared at 100×, and a 2-μl aliquot was then added to wells in columns 1 through 3 (column 1 is a sterile control with no bacteria). The anti-TB compounds were 2-fold serially diluted from columns 3 through 10. At the end of the serial dilution, 100 μl was discarded from column 10. Column 11 is a bacterial control with no anti-TB compound. The MIC/MBC assay plates were then transferred to the BSL3 laboratory.

### MIC determination by MABA.

The frozen bacterial stock was thawed and diluted in 7H12 to attain a bacterial culture concentration of ∼3 × 10^5^ to 5 × 10^5^ cells/ml. An aliquot (100 μl) of this bacterial culture was then inoculated into the assay plates (clear). After 7 days of incubation at 37°C, 20 μl of resazurin dye (0.6 mM) and 12 μl of 20% Tween 80 were added to all of the wells of the assay plate. Fluorescence at 530 nm excitation and 590 nm emission was measured using a Clariostar (BMG Labtech, Ortenberg, Germany) plate reader. The MIC is defined as the lowest concentration that reduced the fluorescence by 90% relative to the bacterial control (58).

### MIC determination by luminescence.

The frozen bacterial stock was thawed and diluted in 7H12 to attain a bacterial culture concentration of ∼3 × 10^5^ to 5 × 10^5^ cells/ml. An aliquot (100 μl) of this bacterial culture was then inoculated into the assay plates (opaque). Luminescence was measured using Centro XS^3^ LB 960 (Berthold Technologies) after 7 days of incubation at 37°C. The MIC is defined as the lowest concentration that reduced the luminescence by 90% relative to the bacterial control.

### MBC determination.

Depending on the CFU data obtained after 3 weeks of incubation, the exact dilution fold for that batch of bacterial culture was decided to get ∼3 × 10^5^ to 5 × 10^5^ cells/ml in the assay well, and luminescence obtained for that dilution fold while freezing stocks was considered as day 0 RLU. Whenever required, cell stock was diluted in 7H12 medium and inoculated in a 96-well microtiter plate. Vial of H37Rv_LuxABCDE cell stock was thawed, sonicated for 10 to 15 s, and then diluted in 7H12 medium. One hundred microliters of bacterial culture was inoculated in columns 2 to 11, from B to G of the assay plate. Growth controls containing no drug were in column 11, and a sterile control without bacteria was in column 1. Cells were plated on 7H11 agar plates for enumeration of *T*_0_ CFU. *T*_0_ RLU for bacterial culture was considered as the luminescence that was taken before freezing of the culture since measuring RLU of the frozen stock did not give the accurate RLU since cells were not metabolically active. Two identical assay plates were prepared and incubated at 37°C; one was used for MBC by CFU measurement on day 7, while the other plate was used for MBC by luminescence.

### By CFU.

On day 7, 3× 200-μl bacterial cultures (since each concentration was in triplicate) from each well was transferred to a 1.5-ml microcentrifuge tube. The supernatant was discarded after centrifugation at 5,000 rpm (2,348 × *g*) for 2 min, and a fresh 600 μl of medium was added. Pellet was resuspended in 7H12 and 10-fold serially diluted for plating 50 μl on 7H11 agar 6-well plates. Agar plates were air-dried for 15 min, sealed with petri-seal tape, and incubated at 37°C for 4 weeks. Colonies were enumerated. MBC is calculated as the concentration at which 99% (2 log scale) killing was observed compared to the day 0 CFU.

### By luminescence.

White opaque Corning 96-well microtiter plates were used for reading luminescence using Centro XS^3^ LB 960 (Berthold Technologies). Luminescence was measured on days 7, 14, and 21. After 14 days, medium from peripheral wells was replaced with a fresh 200 μl of medium. MBC is calculated as the concentration at which a 99% reduction in RLU was observed compared to the *T*_0_ RLU. Detailed protocol for LMBC assay can also be found in reference 59.

### Intracellular activity by luminescence.

Macrophage cell line J774 was maintained and cultured in complete Dulbecco modified Eagle medium (DMEM) (450 ml of DMEM with 55 ml of fetal bovine serum [FBS] and 1 ml of 0.1 mg/ml ampicillin). A frozen vial of J774 cells was thawed in a 37°C water bath and suspended in 14 ml of complete DMEM. The cell culture flask was incubated in a humidified incubator at 37°C and 5% CO_2_ until the cells became confluent. On the day before the infection, 100 μl of 5 × 10^5^ cells/ml culture was seeded in each well of a white, clear-bottom 96-well microtiter plate and incubated in a humidified incubator at 37°C and 5% CO_2_ overnight. On the day of infection, frozen mid- to late log phase culture of M. tuberculosis (H37Rv_LuxABCDE) grown in 7H9 medium was thawed. Bacterial cells were centrifuged (at 10,000 × *g* for 10 min) and washed with complete DMEM three times. The optical density of the bacterial culture was adjusted to 0.015 (∼2 × 10^6^ cells/ml) in a complete DMEM. Bacterial culture was sonicated twice for 10 s to disrupt the cell clumps. J774 cells in 96-well microtiter plates were infected by adding 100 μl of H37Rv_LuxABCDE cell suspension in DMEM. After 2.5 h of infection, the medium was discarded and replaced with 100 μl of complete DMEM containing 100 μg/ml of amikacin. The microtiter plates were then transferred to a humidified incubator at 37°C and 5% CO_2_ overnight. The next day (after 24 h exposure to amikacin), all of the infected wells were washed three times with DMEM, and 100 μl of DMEM with (2-fold serially diluted) or without test compounds was added to the wells. At this time, luminescence was measured (day 0) and cell monolayers were visually inspected under a microscope. The microtiter plates were incubated up to day 7, and luminescence was measured at different time points (day 3, day 5, and day 7). Medium in the 96-well microtiter plate was changed every 2 days (day 2 and day 5) with fresh DMEM containing test compounds. On day 7, after reading the luminescence, 20 μl of resazurin dye solution (0.6 mM in phosphate-buffered saline [PBS]) was added to all of the wells to assess the viability of macrophages and cytotoxicity of compounds against infected macrophages. EC_90_ is defined as the lowest concentration affecting 90% inhibition of luminescence relative to the untreated M. tuberculosis-infected macrophages. Detailed protocol for intracellular activity can also be found in reference 60.

### PAE by luminescence and by CFU.

Outermost wells of a white opaque-bottom 96-well microtiter plate were filled with 200 μl of 7H12 medium. All of the wells in row B (columns 2 to 11) were filled with 100 μl of medium. Wells from 3B to 11G were filled with 180 μl of 7H12 medium. Two microliters of DMSO stocks of compounds were added to the wells in row B to achieve final concentrations of 1× MIC, 10× MIC, and 100× MIC. In each 96-well microtiter plate, 2 compounds at three different concentrations can be tested with two control columns (column 5 and 11). One hundred microliters of H37Rv_LuxABCDE culture was inoculated in row B (column 2 to 11), and plates were placed in the humidified incubator for 3 h at 37°C. The targeted final bacterial inoculum density was ∼1 × 10^6^ CFU/ml. After 3 h of incubation, 20 μl of bacterial cultures from row B was serially 10-fold diluted up to row G, and the plate was transferred back to the incubator. Following serial dilutions, the luminescence of the plate was read every day up to day 14 using a Clariostar (BMG Labtech, Ortenberg, Germany) plate reader. Although, cultures were serially diluted up to 10^−5^, graphs were plotted and PAE was calculated based on the 10^−4^ dilution because plotting the 10^−5^ dilution might have extended the assay beyond 14 days. Detailed protocol for PAE assay can also be found in reference 61.
