# Supplementary material for: In Vitro Profiling of Antitubercular Compounds by Rapid, Efficient, and Nondestructive Assays Using Autoluminescent Mycobacterium tuberculosis
Source: Antimicrob Agents Chemother. 2021 Jul 16;65(8):e00282-21. doi: 10.1128/AAC.00282-21 (PMC8284454; doi:10.1128/AAC.00282-21)
Supplement: Supplemental file 1 — Supplemental material. Download AAC00282-21_Supp_S1_seq4.pdf, PDF file, 0.2 MB [file aac00282-21_supp_s1_seq4.pdf]

In Vitro Profiling of Antimycobacterial Compounds by Rapid, Efficient and Non-Destructive Assays Using Auto-luminescent *Mycobacterium tuberculosis*

Gauri S. Shetye,[a] Kyung Bae Choi,[a] Chang-Yub Kim,[b] Scott G. Franzblau,[a] Sanghyun Cho,[a#]

[a] Institute for Tuberculosis Research, College of Pharmacy, University of Illinois at Chicago, 833 S. Wood Street, Chicago, Illinois 60612, United States

[b] Dept. of Biomodulation, MJ Bioefficacy Research Center, Myongji University, 116 Myongji-ro, Cheoin-gu, Yongin, Gyeonggido, Korea 17058

[#] Address correspondence to Sanghyun Cho, jkcno1@uic.edu.

Supporting information

Table S1. MBC values for ten TB drugs and two emerging (ecumicin, rufomycin) TB leads obtained by LMBCA assay on days 7, 14, and 21. The standard deviation (SD) is from three independent experiments.

**Anti- TB agents**	**MBC (µM) (± SD) by luminescence on**		
	**day 7**	**day 14**	**day 21**
Rifampin	0.6 (0.2)	0.48 (0.10)	0.4 (0.01)
Moxifloxacin	0.91 (0.25)	0.42 (0.02)	0.46 (0.03)
Linezolid	9.6 (2.26)	7.0 (0.15)	6.0 (1.9)
Streptomycin	0.3 (0.05)	0.5 (0.03)	0.9 (0.28)
Capreomycin	1.3 (0.4)	6.94 (3.8)	13.65 (2.34)
Isoniazid	0.57 (0.08)	2.56 (1.2)	3.23 (0.57)
Ethambutol	9.18 (2.7)	3.17 (0.12)	3.28 (0.39)
Pretomanid	0.63 (0.05)	12.5 (0.71)	13 (0.01)
Bedaquiline	0.13 (0.04)	0.11 (0.02)	0.12 (0.06)
Clofazimine	1.81 (0.19)	0.82 (0.08)	1.01 (0.05)
Ecumicin	0.22 (0.1)	0.20 (0.09)	0.12 (0.04)
Rufomycin	> 1	> 1	0.49 (0.01)

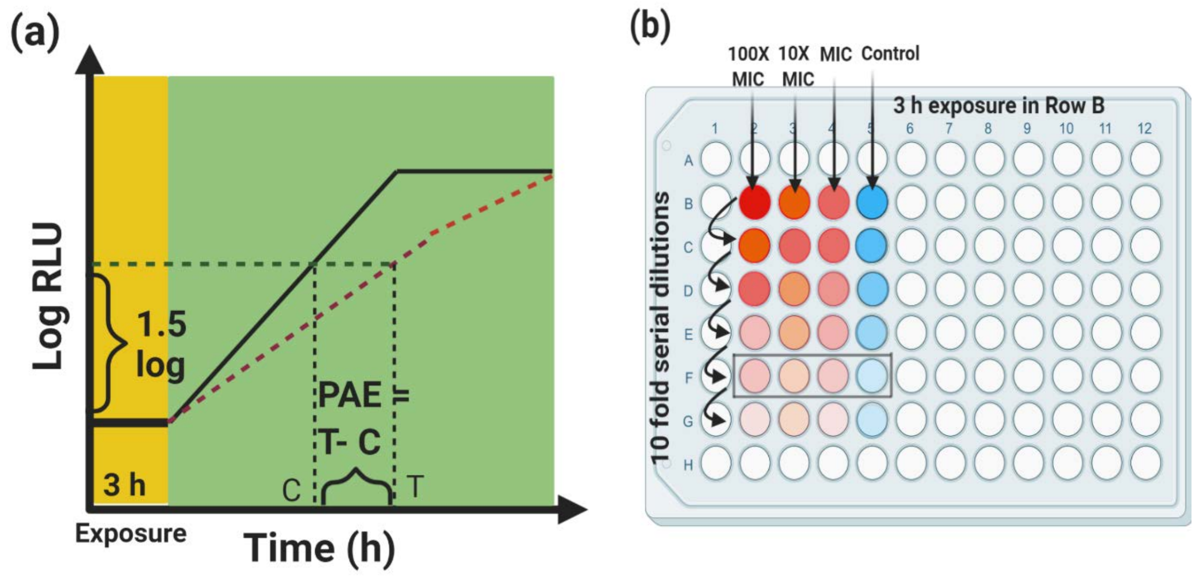

Figure S1. (**a**) PAE was defined as the difference in time (h) taken by the treated culture (T) and bacterial control (C) to exhibit a 1.5 fold increase in log RLU; (**b**) H37Rv_LuxABCDE culture (200 µL) was inoculated in row B along with different compound concentrations (1X MIC, 10X MIC, and 100X MIC). Aliquots of 7H12 media (180 µL) were pre-portioned in rows C through G. After 3 h exposure, 20 µL of treated culture from Row B was transferred to Row C to achieve 10-fold dilution. Similarly, subsequent 10-fold dilutions were serially performed through Row G (1: 100, 000 dilution). PAE growth plots (in days) (**Figure 4**) and PAE values (h) (**Table 4**) were obtained from results associated with 1: 10,000-fold dilutions (Row F).
